# Chemokine Ligand 13 (CXCL13) in Neuroborreliosis and Neurosyphilis as Selected Spirochetal Neurological Diseases: A Review of Its Diagnostic Significance

**DOI:** 10.3390/ijms21082927

**Published:** 2020-04-22

**Authors:** Monika Gudowska-Sawczuk, Barbara Mroczko

**Affiliations:** 1 Department of Biochemical Diagnostics, Medical University of Bialystok, 15-269 Bialystok, Poland; mroczko@umb.edu.pl; 2 Department of Neurodegeneration Diagnostics, Medical University of Bialystok, 15-269 Bialystok, Poland

**Keywords:** CXCL13, chemokine, diagnostic marker, neuroborreliosis, neurosyphilis, inflammation, cerebrospinal fluid

## Abstract

Neuroborreliosis (NB) and neurosyphilis (NS) are abnormal conditions caused by spirochetal bacteria which affect the nervous system. Diagnosis of neuroborreliosis and neurosyphilis is determined by clinical examination of visible symptoms, serum and cerebrospinal fluid (CSF) analysis, and serological detection of antibodies against *Borrelia burgdorferi sensu lato* and *Treponema pallidum,* respectively. Establishing a diagnosis may sometimes pose a number of diagnostic difficulties. A potential role of chemokine ligand 13 (CXCL13) as an accurate diagnostic biomarker of intrathecal inflammation has been suggested. In this review, we focused on changes in serum and cerebrospinal fluid concentration of chemokine ligand 13 in selected spirochetal neurological diseases neuroborreliosis and neurosyphilis reported in the available literature. We performed an extensive search of the literature relevant to our investigation via the MEDLINE/PubMed database. It has been proven that CXCL13 determination can provide rapid information regarding central nervous system inflammation in patients with selected spirochetosis. We described that neuroborreliosis and neurosyphilis are associated with an elevated CXCL13 concentration, mainly in the cerebrospinal fluid. Moreover, literature data suggest that CXCL13 determination is the most interesting additional marker for diagnosis and monitoring of neuroborreliosis and neurosyphilis thanks to its high sensitivity. Based on these published findings, we suggest that CXCL13 has high diagnostic utility and may be applied in laboratory diagnostics as a potential diagnostic marker in human spirochetal neurologic diseases.

## 1. Introduction

Spirochetal diseases are a group of several diseases often characterized by different clinical features and epidemiology, but always caused by spirochetal bacteria. Spirochetes are Gram-negative bacteria which have a unique spiral shape [1]. In this review we focused on two spirochetes: *Borrelia burgdorferi sensu lato* and *Treponema pallidum* that can cause neuroborreliosis (NB) and neurosyphilis (NS), respectively. Both infections often start asymptomatically while the spirochetes multiply. Then the bacteria can disseminate widely, causing inflammation. Both infections elicit systemic B-cell- and T-cell-mediated immune response which is highly focused on the central nervous system (CNS), involving pleocytosis. These abnormal conditions may progress to a chronic infection which can be diagnosed by cerebrospinal fluid (CSF) analysis [2,3,4].

Our review offers an updated analysis of studies, including publications by Halperin and Rupprecht et al., which presents an overview of the diagnosis and management of neuroborreliosis and neurosyphilis and whose authors suggest a potential role of chemokine ligand 13 (CXCL13) as an accurate diagnostic marker of these two spirochetal infections of the nervous system [2,5,6,7,8,9,10,11,12,13]. Chemokines, or chemotactic cytokines, are a very important and large group (more than 50) of low molecular weight proteins structurally homologous to cytokines. Their primary function is to stimulate leukocyte movement and control their migration from the blood to tissues. It has also been proven that chemokines are involved in the activation of adhesion molecules, and regulation of angiogenesis, embryogenesis, organogenesis, and apoptosis [14,15]. Chemokines can be divided into constitutive (lymphoid) and inducible, which are upregulated at sites of inflammation [16,17]. However, classification of chemokines is based on their structure, not their functions. Chemokines are polypeptides composed of 66–111 amino acids. All chemokines have a characteristic tertiary structure stabilized by disulfide bonds between cysteines [18,19]. Based on the number and relative location of the first two cysteines situated at the end of the amino chain, four groups of chemokines can be distinguished: β-chemokines (CC), α-chemokines (CXC), δ-chemokines (CX_3_C), and γ-chemokines (XC). Each subfamily designation is followed by the letter L (as “ligand”) and, at the end, a number used in the encoding gene nomenclature. It is well known that chemokines activate cellular response after binding to a specific receptor. Chemokine receptors are named according to their ligands. The subfamily designation with which the receptor interacts is given, the letter “R” (from the word “receptor”) is added, and a number based on the chronological order in which it was identified is added. Receptor expression on the cell surface can be continuous or the receptor can be revealed on the cell surface after its activation [16,17]. 

The chemokine selected as the focus of our review was first described by Legler et al. in 1998 [20]. Chemokine ligand 13, also known as B-cell attracting chemokine 1 or B lymphocyte chemoattractant, is a member of the CXC chemokine family encoded by the CXCL13 gene located on chromosome 4 (4q21) [20,21]. CXCL13 in humans is mostly produced by dendritic cells, monocytes, and mature macrophages. The chemokine plays an important role in inflammation, infections, and immune response. After binding to the G-protein receptor CXCR5 on neutrophils and mast cells, CXCL13 elicits chemotaxis and migration of B lymphocytes from lymphoid tissues to the site of inflammation. Importantly, it has been suggested that CXCL13 is involved in neurological damage through promoting B cell migration to the CSF and activating them, subsequently leading to CSF pleocytosis with characteristic B cell elevation [12,13,22,23,24,25,26]. Therefore, the aim of this study was to conduct a review of the current literature regarding CXCL13 in serum and CSF to determine its diagnostic significance in spirochetal neurological diseases neuroborreliosis and neurosyphilis.

## 2. Results

**Figure 1:**
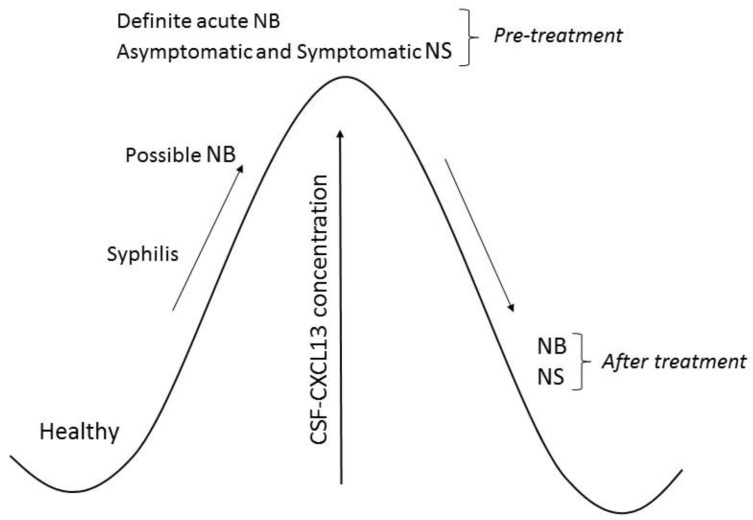
Chemokine ligand 13 (CXCL13) concentration in cerebrospinal fluid (CSF) of patients with neuroborreliosis (NB) and neurosyphilis (NS).

Based on the results published by other authors described below, we created Figure 1 and Table 1. Figure 1 illustrates changes in CXCL13 concentration in the cerebrospinal fluid of patients with neuroborreliosis and neurosyphilis. The diagnostic significance of CXCL13 in neuroborreliosis and neurosyphilis is presented in Table 1.

### 2.1. Neuroborreliosis

Neuroborreliosis is a form of Lyme disease affecting the central and peripheral nervous system. It is caused by infection with the bacterium *Borrelia burgdorferi sensu lato* as a result of a tick bite [57]. Neuroborreliosis is the most commonly diagnosed tick-borne infection of the nervous system in Europe and the United States, affecting 10–15% of infected individuals [27,58]. However, in the United States, neuroborreliosis, isolated or combined with joint symptoms, is less frequent than in Europe [59]. Neuroborreliosis can develop anywhere within the nervous system and is characterized by a variety of symptoms. Besides neurological symptoms, the disease can manifest itself with cardiac, skin, or rheumatic symptoms and it can develop within a few weeks up to several years after infection [59].

#### 2.1.1. Diagnosis

Establishing a diagnosis of neuroborreliosis is associated with many diagnostic difficulties since most symptoms are common for other diseases of the nervous system [60]. Neuroborreliosis requires particularly careful differentiation from multiple sclerosis, neurosyphilis, brain vascular diseases, viral infections, Alzheimer disease, or cancers of the central nervous system [2,28,61]. At present, a diagnosis of neuroborreliosis is based on the patient’s medical history, clinical symptoms of the infection with immunoserological confirmation, or exclusion of the underlying bacterial disease. Blood and CSF are often used in the diagnostic process. In clinical practice, the most commonly utilized methods for *Borrelia burgdorferi sensu lato* identification are serological modalities. Initially, an ELISA test is carried out to detect specific antibodies against *Borrelia burgdorferi sensu lato.* If the ELISA test is positive or inconclusive, a Western blot test should be performed to confirm the reaction with spirochetes. Additionally, a CSF analysis indicates mononuclear pleocytosis and the presence of lymphocytes and monocytes in the smear [29,62,63]. It is also important to understand the difference between diagnoses of definite and possible Lyme neuroborreliosis. Table 2 presents diagnostic criteria for definite and possible Lyme neuroborreliosis according to the European Federation of Neurologic Societies (EFNS) guidelines [64].

Unfortunately, the diagnostic process of neuroborreliosis has numerous limitations and disadvantages. Some laboratory test results may indicate a different, e.g., viral, etiology of the central nervous system dysfunction. Furthermore, CSF tests do not normally reveal any abnormalities when performed within the first two weeks of the infection [65]. Therefore, it is important to find the best diagnostic marker for neuroborreliosis. Early detection of the disease creates a possibility for administering the most appropriate and effective treatment in its early stages. Untreated neuroborreliosis is very dangerous and can lead to irreversible changes in the human body such as dementia or encephalopathy. It is also estimated that <10% of untreated neuroborreliosis cases lead to encephalomyelitis. However, it is worth pointing out that cases of untreated neuroborreliosis and such complications are exceptionally rare today [66,67].

A perfect diagnostic maker should be suitable for early disease diagnosis, useful in determining prognosis, fast, safe, and easy to perform.

#### 2.1.2. CXCL13 Concentration

The pathogenesis of neuroborreliosis involves a complex immune response due to *Borrelia burgdorferi sensu lato*, resulting in the specific activation of B lymphocytes. There is a greater number of activated B cells in the CSF than in the blood, and the percentage of B-cells in the CSF of patients with neuroborreliosis increases up to 80% [47]. One of the key regulators of B-cells is CXCL13, whose concentration may be elevated at the onset of neuroborreliosis, before the synthesis of intrathecal *Borrelia burgdorferi sensu lato* antibodies occurs [47]. Several studies have demonstrated a strong association between CXCL13 and specific neuroinflammatory diseases when compared to non-inflammatory central nervous system disorders or asymptomatic HIV infection. However, most studies measuring CXCL13 using ELISA and lateral flow (LFA) methods have shown elevated CXCL13 circulation levels in the CSF of patients with acute neuroborreliosis compared to other tested groups. Results of the available studies suggest its very high diagnostic value and report its high sensitivity and specificity [5,6,7,27,28,29,30,31,32,33,34,35,36,37,38,39,40,41,42,43,44,45,46]. These studies are in agreement with a meta-analysis performed by Yang J. et al., who confirmed that CXCL13 has a high sensitivity and specificity for diagnosing neuroborreliosis [68].

Based on the pooled specificity, sensitivity, positive likelihood ratio, negative likelihood ratio, diagnostic odds ratio, and the receiver operating characteristic curve, we found that CXCL13 has high diagnostic value, which means that it can be used as a new biomarker in the diagnosis of Lyme neuroborreliosis.

Moreover, it has been proven that CSF levels of CXCL13 are significantly increased in patients with definite acute neuroborreliosis, possible neuroborreliosis with pleocytosis, and possible neuroborreliosis with positive specific antibodies. However, CXCL13 levels in patients suffering from possible neuroborreliosis with positive specific antibodies have been found to be the lowest, which may reflect a previous infection or different, not associated with neuroborreliosis, causes of the presenting symptoms. It also may indicate that CXCL13 correlates better with pleocytosis than with the CSF-specific antibodies index. Such results also suggest that CXCL13 levels can be helpful in distinguishing the activity and acuteness of the current infection from previous infections [5,34,37,38,39,40]. Levels of the chemokine in possible neuroborreliosis with negative specific antibodies has been found not to differ from other neurological conditions, e.g., multiple sclerosis, viral meningitis, neurosyphilis, or lupus [41].

Semel et al. focused on CXCL13 as a potential indicator of neuroborreliosis activity. The major finding of the study was that patients with shorter disease duration prior to a lumbar puncture showed significantly increased CXCL13 concentrations in comparison to patients with longer disease duration. Higher CXCL13 levels in the latter group of patients were probably associated with a higher leukocyte count in CSF. The relationship between CXCL13 concentration and a white blood cell count confirms that CXCL13 correlates with inflammatory disease activity and accumulation of polyspecific intrathecal B cells [41,42].

#### 2.1.3. Treatment and CXCL13 Concentration

It is known that CXCL13 plays a functional role in the development of various neurological diseases. Elevated levels of this B-cell chemoattractant are known to be associated with neurogenesis, brain development, neuromodulation, and inflammation of the central nervous system. The impact of neuroborreliosis treatment on CXCL13 expression has been evaluated in patients treated with, e.g., doxycycline [7,28,32,33,36,43]. Recent studies have demonstrated that serum and CSF CXCL13 concentrations were significantly increased at disease onset and prior to treatment commencement in comparison to the control group. Following treatment, CSF CXCL13 decreased in neuroborreliosis (e.g., 3.2-fold in a study by Moniuszko et al. [43]), but the concentration was still higher in comparison to the control group, where it was undetectable. Repeated CXCL13 measurements, mainly in the CSF, might be an attractive diagnostic marker of treatment response since treatment with anti-*Borrelia burgdorferi-*specific antibiotics has been shown to result in a significant reduction in the concentration of this chemokine. The authors suggest that CXCL13 should be combined with other diagnostic indicators of disease activity and treatment response [43].

#### 2.1.4. CXCL13 in Pediatric Patients

Studies on the diagnostic utility of CXCL13 measurement concern mostly adults, but there are some studies involving younger patients. The aims of pediatric studies included a reduction in mortality rates and control of the spread of infectious diseases. These studies have proven that CXCL13 concentration in the CSF of pediatric patients is significantly increased in neuroborreliosis compared to non-neuroborreliosis individuals [8,9,44,47]. One study revealed that differences in CXCL13 levels between children with neuroborreliosis and those with other conditions were statistically significant, although relatively small [45]. CXCL13 also confirmed the diagnosis of possible neuroborreliosis in 100% of patients, indicating a higher diagnostic sensitivity than intrathecal-specific antibodies synthesis [27,48]. Moreover, CXCL13 appears to be detectable earlier than antibodies against *Borrelia burgdorferi sensu lato* [50]. All children with possible neuroborreliosis, pleocytosis, facial nerve palsy, and/or anti-*Borrelia burgdorferi sensu lato* antibodies have been found to have increased levels of CXCL13 in the CSF [8,47]. Some authors have demonstrated that serum and CSF CXCL13 levels are elevated in all patients (adults and children) with definite acute neuroborreliosis [27,48]. Furthermore, some studies have investigated differences between CXCL13 concentrations in adult and pediatric patients and revealed that CXCL13 levels are lower in children, but the difference is not significant [9]. Therefore, we believe that the issue of CXCL13 concentration differences between children and adults should be examined on a larger cohort.

In recent years, new diagnostic markers have been tested in pediatric patients and compared to current tests, which are a component of patient classification procedures. Combining CXCL13 with other diagnostic markers for neuroborreliosis has produced promising results. A study by Skogman et al. explored whether early diagnostic markers including CXCL13, IgM CSF/serum index, and anti-Borrelia antibody tests could be useful as complementary tools for diagnosing neuroborreliosis in children. Among pediatric patients with definite neuroborreliosis, 89% tested positive with the recomBead Borrelia assay. Of the studied patients, 97% tested positive for CXCL13 in CSF, whereas the sensitivity of IgM index came to 84%. However, the authors hypothesized that since all diagnostic markers differed significantly in patients with neuroborreliosis compared to those with other diseases, it may be an indication to check the diagnostic utility of these marker combinations. For a positive combined test result, children needed to have a positive recomBead Borrelia antibody test and a positive CXCL13 or IgM index. Interestingly, in the neuroborreliosis group, 100% of the studied patients tested positive when a combination of tests was used [44]. Another study evaluated, in parallel, a combination of CSF-serum CXCL13 ratio and CSF-C6 antibodies. However, the authors did not observe significant differences between the AUC for CSF-serum CXCL13 ratio and that for a combination of CSF-serum CXCL13 ratio and CSF-C6 antibodies [46]. Therefore, combining CXCL13 with other available laboratory tests is still questionable.

### 2.2. CXCL13 in Neurosyphilis

The World Health Organization estimates that every year 11 million new cases of the disease are diagnosed among people aged 15–49 years old globally. Syphilis is a chronic infectious disease transmitted by sexual contact or transplacentally in utero from an infected mother to the fetus. It is an infection caused by the bacterium *Treponema Pallidum.* Neurosyphilis is caused by an invasion of the spirochetes, which, firstly, attack the meninges and then the parenchyma of the central nervous system or the spinal cord [69,70].

#### 2.2.1. Diagnosis

It is commonly known that diagnosing neurosyphilis is still a complex clinical problem. A reduction in the number of preventive serological tests performed contributes to an increase in the number of cases of syphilis. Abandonment of preventive examinations means that in many patients the disease is undiagnosed. Undiagnosed early syphilis is the reason for an increased incidence of symptomatic syphilis or neurosyphilis [49,71]. Diagnosis of neurosyphilis is established on the basis of clinical signs and laboratory diagnostic tests of serum and CSF. Pleocytosis (corresponding to symptoms), an increased total protein concentration, and a normal or decreased glucose level in the CSF are usually observed. The condition also sometimes results in elevation in gamma-globulins concentration and the presence of oligoclonal bands in the CSF. Diagnosis of neurosyphilis also requires a positive result of the Venereal Disease Research Laboratory (VDRL) serological test. The VDRL test has been found to be highly specific, but blood contamination of CSF must be avoided since it may produce false-positive CSF results [72,73].

#### 2.2.2. CXCL13 Concentration

Neurosyphilis is demonstrated by the presence of an increased number of human B-cells and an abnormal B cell response in the CNS, and it is well known that CXCL13 is regarded as B cell attracting chemokine 1 [51]. A potential role of CXCL13 in neurological damage has been suggested due to its selective chemotactic activity for B lymphocytes, thereby regulating inflammation in the nervous system [10,51]. Therefore, chemokine ligand 13 has been examined and strongly indicated as a possible diagnostic tool in the diagnosis of neurosyphilis. Recent retrospective studies have revealed that CXCL13 levels are increased in the CSF and serum of patients with early and late neurosyphilis. Those patients were found to have higher CSF levels of the chemokine than individuals without neurological symptoms [6,10,11,12,51,52,53,54,55,56]. Release of high levels of CXCL13 during the in vitro incubation of monocytes with *Treponema pallidum* may be evidence that CXCL13 could be a good diagnostic marker for nervous system infections caused by this particular spirochete [10]. As an example, to differentiate neurosyphilis from other neurological disorders, a cut-off point of 76.3 pg/mL of CXCL13 has been suggested with a sensitivity and specificity of 50% and 90%, respectively [52]. It is well known that the closer the AUC is to 1, the higher the sensitivity of the test is to identify disease presence. However, each study suggests different cut-off points and the best CXCL13 cut-off levels are still under debate.

Recent studies have demonstrated that CXCL13 in the CSF positively correlates with total protein concentration, white blood cells count, IgG index, IL-6 and IL-10, and negatively with IL-12. It may indicate the interaction between chemokines and cytokines in neurosyphilis. Additionally, there is no correlation between serum and CSF concentration of CXCL13, which may suggest that elevated levels of the chemokine in the CSF are the result of enhanced synthesis in cells of the nervous system, but not in peripheral blood [56].

Other studies have compared CXCL13 concentrations in neurosyphilis and other CNS conditions. Higher concentrations of CSF CXCL13 have been observed in symptomatic neurosyphilis in comparison to asymptomatic neurosyphilis. In contrast, serum CXCL13 levels have been found to be elevated in asymptomatic neurosyphilis [53]. In regard to CSF CXCL13 concentration and its usefulness in differentiating between neurosyphilis and neuroborreliosis, data are inconsistent. Dersch et al. revealed no differences between neurosyphilis and neuroborreliosis, whereas Rupprecht et al. and van Burgel et al. demonstrated higher concentrations of CXCL13 in neuroborreliosis [6,9,10,12]. Our review demonstrates that this issue is still uncertain and should be investigated further. At the same time, the highest concentration levels of CXCL13 have been observed in patients with neurosyphilis, meningoencephalitis, or encephalomyelitis in comparison to neurosyphilis patients with cranial nerve palsies and cerebral luetic vasculitis [6].

Interestingly, Zeng et al. [11] calculated Q_CXCL13_ using a combination of serum and CSF concentration of CXCL13 and albumin. The Q_CXCL13_ value was significantly increased in neurosyphilis in comparison to syphilis and other central nervous system disorders. The cut-off point of Q_CXCL13_ indicated by the authors showed 87.5% sensitivity and 69.2% specificity, which may suggest that Q_CXCL13_ is a good diagnostic marker to discriminate neurosyphilis from syphilis and other neurological diseases [11]. Furthermore, some studies have demonstrated that the CSF/serum ratio is significantly increased in neurosyphilis patients [12,55]. Rupprecht et al. observed that in 100% of neurosyphilis patients the ratio was low (below 4), whereas in 89.5% of neuroborreliosis patients, the ratio was above the cut-off value. Therefore, the CSF/serum ratio may be useful in differentiating neurosyphilis from neuroborreliosis [12].

Along with CXCL13, Wang et al. analyzed the expression of 35 other chemokines in the CSF and serum of patients with neurosyphilis and found that the levels of CXCL13, CXCL10, and CXCL8 were markedly elevated in those individuals. The concentrations of the studied chemokines also correlated with VDRL CSF titer and CSF total protein concentration. Interestingly, combining multiple markers enhanced test accuracy. A combination of three chemokines was positively correlated to a high AUC value (0.949), 90.4% sensitivity, and 92.9% specificity. However, it was not markedly different from the high diagnostic significance of CXCL13 or VDRL [55]. Therefore, CXCL13 may be a good diagnostic marker in situations in which CSF-VDRL results are nonreactive [53].

Several infections of the central nervous system, including HIV infection, cause abnormalities in humoral immunity. Additionally, HIV infection can promote *Treponema pallidum* infection. Establishing a diagnosis of asymptomatic neurosyphilis in HIV-infected patients poses more difficulties. Therefore, some authors have focused on patients who were co-infected with HIV and neurosyphilis and attempted to investigate CXCL13 levels in these patients. It has been demonstrated that patients with neurosyphilis and HIV co-infection have higher serum concentrations of CXCL13 than HIV-infected patients with syphilis, but without neurological symptoms [52,53]. Zeng et al. suggested that serum CXCL13 levels are not useful in support of the diagnosis of neurosyphilis in HIV-negative patients, since serum CXCL13 concentrations are similar in neurosyphilis and other neurological viral or cryptococcal infections [11]. However, patients with syphilis and HIV co-infection have been found to have higher CSF CXCL13 values than those with HIV infection only [49]. Moreover, CSF CXCL13 levels have been shown to be significantly higher in symptomatic and asymptomatic neurosyphilis in comparison to uncomplicated syphilis [52,53]. The concentration of the chemokine has been found to be lower in HIV-positive/asymptomatic neurosyphilis than in HIV-negative/asymptomatic neurosyphilis, but the differences are not significant [54]. In patients without neurosyphilis, no significant differences between HIV-positive and HIV-negative infection have been revealed [52]. Furthermore, the odds of symptomatic neurosyphilis with HIV co-infection increase significantly with a rise in CSF CXCL13 levels, white blood cell count, detection of *Treponema pallidum,* and plasma HIV RNA. By contrast, the odds of symptomatic neurosyphilis correlate negatively with an increased blood CD4+ T lymphocytes count [52,53]. However, one study indicated that there was no correlation between CSF CXCL13 levels and CD4+ T-cell count in syphilis and HIV co-infection [54]. Summarizing, human immunodeficiency virus provokes an early profound response of B lymphocytes in the CSF, which are the main source of CSF CXCL13 elevation [52].

#### 2.2.3. Treatment and CXCL13 Concentration

CXCL13 in CSF has been assessed as a potential diagnostic marker of an antibiotic treatment response. Therefore, some researchers have measured the concentration of this chemokine prior to and following neurosyphilis treatment [6,53,54,55,56]. Interestingly, before treatment began, CXCL13 showed a correlation with a serum rapid plasma reagin (RPR) test, which measures the concentration of antibodies typically produced in neurosyphilis [55]. Within a few months (e.g., 3, 6, 12) of treatment commencement, a marked decline in CXCL13 concentration was observed, comparable to pretreatment values [6,53,54,55]. Following treatment for neurosyphilis, CXCL13 declined rapidly in parallel with a WBC count and improvement in clinical symptoms [6]. These results indicate that CXCL13 may be used to evaluate the effectiveness of anti-*Treponema pallidum* treatment in patients with neurosyphilis [6,53,54,55].

### 2.3. Limitations

In the present review we focused on studies published in English within the last decade, which could have resulted in some information not being included in the paper. This review demonstrates that the presence of CXCL13 in CSF is not unique to neuroborreliosis or neurosyphilis. In addition, CXCL13 measurements in the blood are not specific to neuroborreliosis and neurosyphilis patients, since CXCL13 concentrations in healthy individuals and other CNS disorders are often comparable to those in subjects with neuroborreliosis and neurosyphilis. Therefore, a lumbar puncture, which is one of the procedures routinely performed to diagnose serious infections of the nervous system, cannot be replaced. It should also be pointed out that increased levels of this chemokine can be observed in most diseases with CSF pleocytosis. Moreover, research studies on the diagnostic significance of CXCL13 in neuroborreliosis and neurosyphilis were performed on relatively small, heterogeneous cohorts. Therefore, comparative studies on larger sample sizes should be conducted to improve the quality and accuracy of results.

## 3. Methods

### Literature Search and Data Extraction

**Figure 2:**
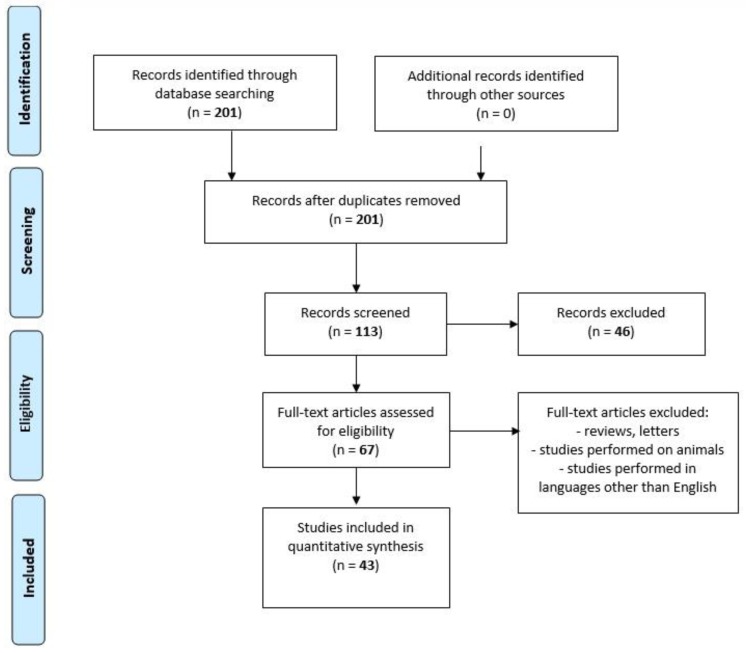
PRISMA flow diagram modified from Liberati et al. [74].

We performed a comprehensive literature search covering the period up to February 2020 using the MEDLINE/PubMed electronic database with the following search strategy: Key words, “chemokine AND spirochetosis” (201 studies). When we used the key words “chemokine AND neuroborreliosis”, a total of 95 papers were found. A search including the key words “chemokine AND neurosyphilis” produced a total of 18 papers. By adding “AND CXCL13” to the key words mentioned above, we limited the search to articles regarding chemokine CXCL13 in neuroborreliosis (*n* = 52) and in neurosyphilis (*n* = 15). The next step involved limiting the search to human studies written in English. Following that, the search was narrowed down to research studies published within the last 10 years. In the final step, we excluded all letters to the editor and review papers. Thus, 31 original publications on CXCL13 in neuroborreliosis and 12 original papers on CXCL13 in neurosyphilis were included in the study (Figure 2, PRISMA flow diagram modified from Liberati et al. [74]).

## 4. Conclusions

The pathogenesis of human spirochetosis involves a complex immune response to *Borrelia burgdorferi sensu lato* and *Treponema pallidum,* resulting in the specific activation of B lymphocytes. Because of the fact that one of the key regulators of B-cells is chemokine CXCL13, a number of research papers evaluating the diagnostic significance of CXCL13 in patients with neuroborreliosis and neurosyphilis were presented in this review. Based on these published findings, we suggest that CXCL13 determination in CSF improves the sensitivity of neuroborreliosis and neurosyphilis diagnostic tests, and supports the implementation of CXCL13 determination in routine laboratory diagnostics for both infectious diseases. CXCL13 may serve as an additional, potential diagnostic marker for diagnosing of selected spirochetal diseases of the central nervous system.

## Authors Contributions

M.G.-S. and B.M. produced the idea of the study and analyzed the data. All authors have read and agreed to the published version of the manuscript.

## Figures and Tables

**Table 1:** Significance of Chemokine ligand 13 (CXCL13) as a candidate diagnostic marker for neuroborreliosis (NB) and neurosyphilis (NS).

	Results	References
Neuroborreliosis	CSF CXCL13 levels are highly elevated in NB compared to healthy and other non-inflammatory CNS diseases	[5,6,7,27,28,29,30,31,32,33,34,35,36,37,38,39,40,41,42,43,44,45,46]
	CSF CXCL13 levels are significantly elevated in NB in comparison to NS	[9,10,12]
	CSF CXCL13 concentrations are elevated in acute and possible NB with positive specific antibodies against *Borrelia burgdorferi*	[5,34,37,38,39,40]
	CSF CXCL13 concentrations correlate with WBC count and disease activity	[41,42]
	CSF CXCL13 concentrations correlate better with pleocytosis than with CSF-specific antibodies	[5,34,37,38,39,40]
	CSF CXCL13 levels are highly elevated in NB patients with shorter disease duration	[41,42]
	CSF CXCL13 levels are markedly elevated before treatment compared to after treatment	[7,28,32,33,36,43]
	CSF CXCL13 levels in pediatric patients with NB are significantly elevated compared to non-NB patients	[8,9,44,45,47]
	CSF CXCL13 has higher diagnostic sensitivity than intrathecal specific antibodies against *Borrelia burgdorferi* and IgM CSF/serum index	[5,27,48,49]
	CSF CXCL13 is detectable earlier than specific antibodies against *Borrelia burgdorferi*	[50]
	Combination of CXCL13, specific antibodies against *Borrelia burgdorferi* and IgM CSF/serum index correctly identify 100% of pediatric patients with neuroborreliosis	[44]
	Combination of CSF-serum CXCL13 ratio and C-6 peptide has similar sensitivity and specificity as CSF-serum CXCL13 ratio alone	[46]
Neurosyphilis	Serum and CSF CXCL13 levels are increased in early and late NS compared to other CNS disorders and syphilis	[6,10,11,12,51,52,53,54,55,56]
	CSF CXCL13 positively correlate with total protein, IL-6, IL-10, IgG index and VDRL CSF titer	[55,56]
	CSF CSF CXCL13 negatively correlate with IL-12	[56]
	CSF CXCL13 levels are higher in symptomatic than asymptomatic NS	[55]
	CSF CXCL13 levels are similar in NS and NB	[6]
	Q_CXCL13_ is significantly elevated in NS than in other CNS disorders	[11]
	CXCL13 CSF/serum ratio is increased in NS	[12,55]
	CSF CXCL13 levels are markedly elevated before treatment compared to after treatment	[6,53,54,55,56]
	Serum CXCL13 is markedly elevated in NS and HIV co-infection compared to HIV infection only	[52,53,54]
	CSF CXCL13 concentrations correlate with WBC count, CSF-specific antibodies against *Treponema. pallidum* and plasma HIV RNA	[52,53]
	CSF CXCL13 levels correlate negatively with CD4+ lymphocytes count	[52,53]

**Table 2:** Diagnostic criteria for definite and possible Lyme neuroborreliosis according to the European Federation of Neurologic Societies (EFNS) guidelines.

Lyme Neuroborreliosis	
Definite	Possible
Three criteria fulfilled	Two criteria fulfilled
Neurological symptoms suggestive of Lyme neuroborreliosis without other obvious reasons CSF pleocytosis Intrathecal *Borrelia burgdorferi* antibody production	

CSF; cerebrospinal fluid.
